# Trials evaluating drug discontinuation: a scoping review sub-analysis focusing on outcomes and research questions

**DOI:** 10.1186/s12874-025-02597-z

**Published:** 2025-05-27

**Authors:** Nele Kornder, Norbert Donner-Banzhoff, Ina Staudt, Nina Grede, Annette Becker, Annika Viniol

**Affiliations:** 1 Department of Primary Care, University of Marburg, Karl-Von-Frisch-Straße 4, Marburg, 35043 Germany; 2 Department of Primary Care, Goethe University Frankfurt, Theodor-Stern-Kai 7, Frankfurt Am Main, 60590 Germany

**Keywords:** Polypharmacy, Deprescribing; Drug discontinuation, Scoping Review, Outcome Selection

## Abstract

**Background:**

The widespread use of long-term pharmacological treatments for chronic conditions has led to polypharmacy, raising concerns about adverse effects and interactions. Deprescribing, the discontinuation of drugs with unfavorable benefit-risk ratios, is gaining attention. Studies evaluating the discontinuation of drugs have a broad methodological spectrum. The selection of outcomes poses a particular challenge. This scoping review addresses the methodological challenges of outcome selection in RCTs investigating drug discontinuation.

**Methods:**

The scoping review includes RCTs that investigated the discontinuation of drugs whose efficacy and/or safety was in doubt. Data on study characteristics, the motivation for evaluating drug discontinuation, the number and type of primary endpoints, and the stated hypotheses were extracted and analyzed.

**Results:**

We included 103 RCTs. Most studies were from Europe and the USA and mainly investigated antipsychotics/antidepressants, immunosuppressants, steroids and antiepileptics. The discontinuation studies were often conducted due to side effects of the treatment and doubts about the benefits of the drug. The primary endpoints reflected either the course of the disease ("justification of treatment") or the disadvantages of the drug ("justification of withdrawal”). Non-inferiority hypotheses were generally prevalent in justification of treatment studies, while superiority hypotheses were more commonly used in justification of withdrawal studies. However, due to methodological and practical challenges this was not always the case.

**Conclusion:**

We present a framework to choose outcomes and specify hypotheses for discontinuation studies. With regard to this, both key challenges (justification of treatment and justification of withdrawal) must be met.

**Supplementary Information:**

The online version contains supplementary material available at 10.1186/s12874-025-02597-z.

## Introduction

### Rationale

Many chronic conditions that were once treatable only symptomatically are now amenable to long-term pharmacological therapy, improving patients’ long-term prognosis. Randomized controlled trials (RCTs) have established a robust evidence base for the safe and effective treatment of chronic diseases, such as heart failure [1], asthma [2] or rheumatoid arthritis [3]. Nevertheless, these improvements have contributed to a growing concern: polypharmacy. Since the prevalence of most chronic conditions increases with age [4, 5], older persons are particularly affected, often receiving multiple concurrent prescriptions [6]. Even when each drug is evidence-based and appropriate on its own, their combination may lead to adverse effects, drug interactions, and an increased risk of medication errors [7]. Indeed, adverse drug reactions represent a major cause of hospital admissions [8, 9]. Polypharmacy has become an urgent topic for health policy, practice and research [10].

Discontinuing drugs that no longer offer a favorable benefit–risk ratio for the individual patient appears to be a logical response to this dilemma.

However, scientific evidence supporting this approach remains limited—especially when compared to the extensive research on initial drug prescribing. Concerns about potential harms, such as withdrawal symptoms or disease recurrence, further contribute to uncertainty and may impede the discontinuation of drugs in practice [11].

Clinicians considering drug discontinuation and researchers studying this process face two main challenges: 1) treating the underlying condition for which the drug was prescribed, and 2) addressing the potential disadvantages of the treatment. While the latter motivates critical evaluation of the drug, the former must be carefully considered to avoid harm from under-treatment. Achieving a high drug discontinuation rate, whether in clinical practice or research, is not a success in itself. For instance, if patients experience harmful consequences such as disease flare-ups after discontinuation, the results of such studies may be ambiguous, and the implications for future care may be unclear. Thus, drug discontinuation studies often raise complex questions that extend beyond those typically addressed in studies of drug safety and efficacy.

To gain a deeper understanding of the complexities of discontinuation studies, our research group has developed a typology that categorizes the different scientific questions related to drug discontinuation and offers type-specific methodological recommendations [12]. Among these, type 1 research questions, which concern doubts about the effectiveness and/or safety of a drug, are the focus of this paper.

### Objectives

The selection of appropriate outcomes is crucial in this context, as it must align with the motivations for discontinuation while addressing both the challenges of treating the condition and evaluating the potential disadvantages of the treatment. Based on a systematic review of studies evaluating drug discontinuation [13], we analysed a subset of RCTs included in this paper. Our objectives were to: 1) assess the authors'motivations for conducting drug discontinuation studies, 2) examine how they addressed the dual challenges of treatment efficacy and discontinuation disadvantages, and 3) explore the outcomes they selected to evaluate these issues. Throughout the paper, we use the terms “drug withdrawal” and “drug discontinuation” interchangeably.

## Methods

### Eligibility criteria

In this scoping review, we identified studies on drug discontinuation, irrespective of the specific drug, disease, or outcome. For this analysis, we used a subset of the review by Grede et al. [13], consisting exclusively of RCTs classified as type 1 drug discontinuation studies, according to Viniol et al. [12]. These studies focus on discontinuation due to concerns about the efficacy or safety of continued drug therapy.

We included only original studies with a prospective RCT design. RCTs provide the highest level of evidence for evaluating the effects of drug discontinuation in clinical practice and represent the methodological standard that future discontinuation studies should adhere to. Eligible studies examined the discontinuation of at least one drug, focusing either on a single agent or one from the same pharmacological class. Type 2 and type 3 studies, which evaluate structured discontinuation procedures or complex discontinuation strategies, were excluded.

We also excluded case reports with only one participant, studies that did not involve humans, and studies that have not been published in English, German, Spanish, or French. Retrospective studies were not included to ensure methodological consistency and avoid bias related to data collection and confounding variables. Observational studies, including cohort and case–control studies, were excluded to maintain comparability across trials.

Furthermore, studies investigating on-demand medications were not considered, as these do not reflect continuous pharmacological treatment. Trials in which a drug was introduced and then discontinued as part of the study to demonstrate efficacy were also excluded, as these studies do not align with the aim of evaluating the discontinuation of established long-term therapies. Finally, we excluded studies focused on substitution therapies in the context of substance abuse or addiction due to their distinct clinical and methodological nature.

### Information sources and search strategy

The dataset was obtained following the systematic search strategy developed by Grede et al. [13]. The search encompassed multiple databases, including Medline (PubMed), The Cochrane Library, EMBASE, CINAHL, Web of Science, and PsycINFO. The original search was conducted in January 2016 and subsequently updated in March 2021 to include all references published up to the end of 2020. Search terms comprised “discontinuation” along with relevant synonyms, as well as the MeSH terms “Safety-Based Drug Withdrawals” and “Drug Therapy.” A comprehensive description of the search methodology, including detailed examples for Medline, is available in the Supplementary Material of our previous publication [13].

### Data charting process

To describe the sample, we extracted bibliographic data, including funding information, from all included studies (see Table 1). The investigated drugs were classified into distinct pharmacological groups (see Table 1). A pharmacological group was then redefined when medications from at least two studies could be assigned to it. This led to the creation of the category"Other drugs for chronic conditions", which includes pharmacological subclasses that appeared only once in the sample. An overview of the subclasses can be found in Table 1.

We extracted information regarding the motivation for drug discontinuation, primary outcomes, and study hypotheses. Initially, the original wording was documented and then summarized and categorized. Categories were iteratively developed and validated by the study team. Data extraction was performed by IS, with ambiguous cases discussed with NDB, AV, and NK.

### Key data items and synthesis of results

We extracted and analyzed key aspects of each included study to understand how drug discontinuation was conceptualized and evaluated.

#### Motivation for evaluating drug discontinuation

We first identified the “initial therapeutic objective” of each drug, as specified by the authors (e.g., cure, prophylaxis). Subsequently, we grouped the reported reasons for drug discontinuation into predefined categories, such as “side effects” and “doubts about the benefits of the drug.” A complete list of these categories and their definitions is provided in Table 2.

#### Primary outcomes

We recorded whether the primary outcome was explicitly defined, could be inferred from contextual information (e.g., sample size calculation), or was not discernible. The category “structure of primary outcome(s)” differentiates between single outcomes, composite outcomes (i.e., the endpoint occurs if any of several events occur, such as death, myocardial infarction, or stroke), co-primary outcomes (i.e., two or more distinct endpoints must be met), and multiple outcomes (neither co-primary nor composite) (see Table 3).

The category “type of outcome” classifies primary outcomes as either clinical (e.g., symptoms, quality of life, mortality), subclinical (e.g., biochemical test results, imaging findings), or a combination of both. In addition, the discontinuation rate was included in this category. The discontinuation rate is defined as the proportion of patients who resumed medication following an initial discontinuation. We also categorized the primary outcome based on the clinical challenge it addresses: whether it aims to justify continued treatment (Justification of Treatment, JT), justify withdrawal (Justification of Withdrawal, JW), or both. Finally, we categorized the role of"time"in defining the endpoint (e.g., number of events during follow-up or time to event). Further details and definitions of the categories of interest can be found in Table 3.

#### Study hypotheses

In the first step, we mapped the study hypotheses—when explicitly stated by the authors—to current definitions of “superiority” or “non-inferiority” designs [14]. This classification was only possible when the hypothesis was clearly stated, which was not always the case. Therefore, we created the category “no specification in the text” for studies in which the hypothesis was either not identifiable or not mentioned. In the second step, we evaluated the hypotheses or research questions based on their context and clinical relevance, which led to the category “appropriate hypotheses according to us.” For this classification, two authors (IS, NDB) independently assessed and categorized the hypotheses of the included studies as either superiority or non-inferiority. Each author conducted the classification independently. In cases of agreement, the categorization was accepted; in cases of disagreement, a third author (AV) reviewed the study and made the final decision (see Table 4).

### Statistical analysis

We calculated descriptive statistics (frequencies and percentages) for each extracted category. All statistical analyses were performed with SPSS software (v 22.0; IBM Corporation, Armonk, NY, USA).

## Results

### Study selection

**Fig. 1:**
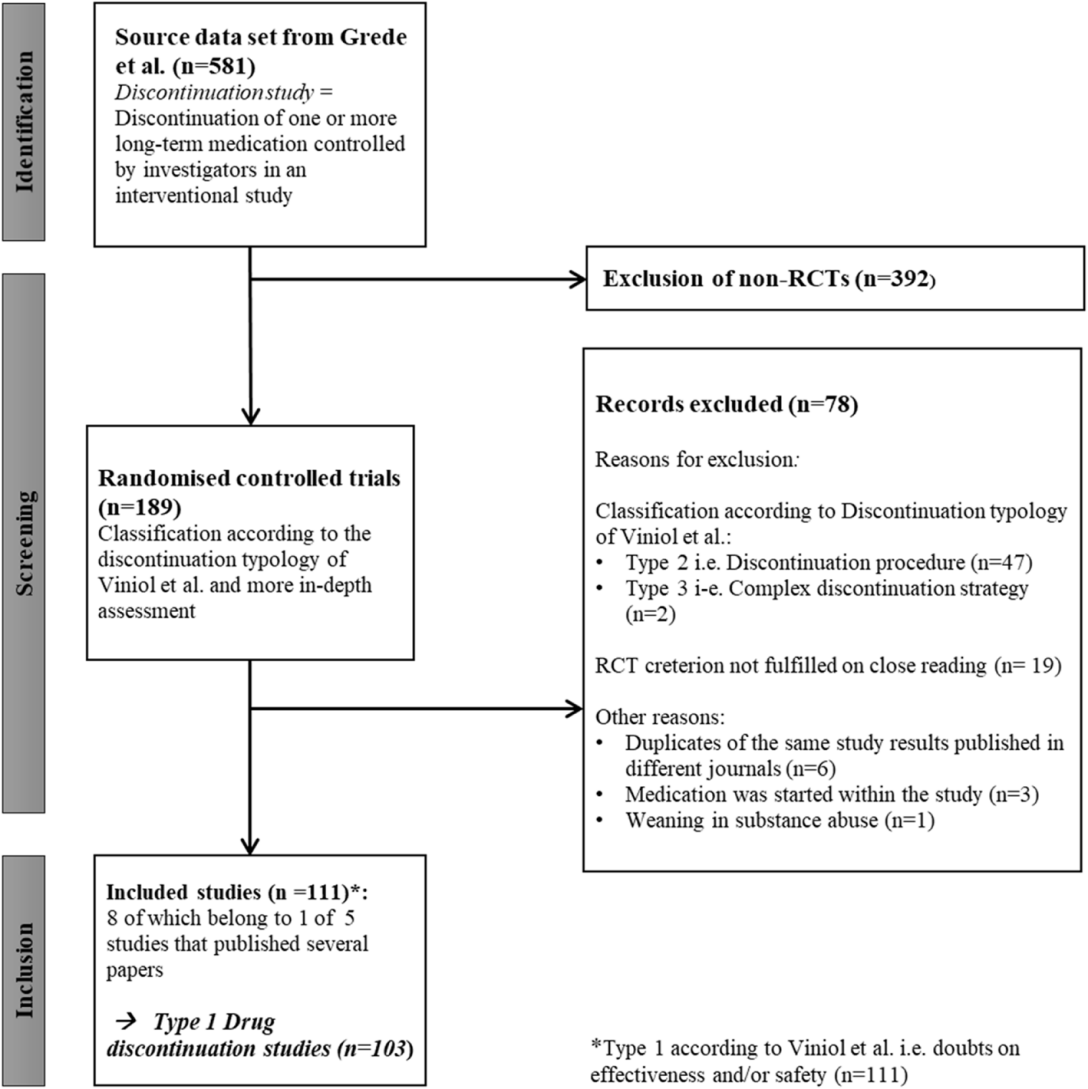
PRISMA flow diagram [15]

The scoping review by Grede et al. [13] identified 581 discontinuation studies. Among them were 189 RCTs, of which 103 were type-1 drug discontinuation studies. Finally, 111 articles were selected for analysis, eight of which are associated with one of five studies that published multiple papers contributing to the analysis presented here (see Fig. 1). The numbers of records identified, screened, excluded, and included are shown in the PRISMA—ScR flow diagram [15] (Fig. 1).

### Characteristics of included studies

The majority of the studies we investigated were from Europe (62.1%) and the USA (21.4%). These studies have been published since the late 1970 s, with a peak between 1990 and 2009 (61.1%) (see Table 1). In total, we identified and grouped the discontinued substances into 12 distinct drug classes. The 103 included trials evaluated drugs from a range of pharmacological classes, with the most common being immunosuppressants (18.4%) and antipsychotics (20.4%). An overview of the drug classes can be found in Table 1.

In 58 studies (56.3%), the discontinuation of a single, prespecified drug was investigated (e.g., lithium for the treatment of depression [16]). In 39 studies (37.9%), the discontinued medication was selected from a drug class—such as antiepileptic drugs (AEDs), including carbamazepine, valproate, phenytoin, phenobarbital, or lamotrigine [17]. In all included studies, only one drug was discontinued per patient, with the only exception being the discontinuation of fixed drug combinations for infection prophylaxis [18]. In six studies, it was not possible to determine the exact number of substances discontinued, as only general drug class labels were provided (e.g., sulfonylureas [19]*,* proton pump inhibitors [20]), without specification of the individual agents.

For a comprehensive listing of all included studies along with their characteristics, please refer to Supplementary Table 1.

**Table 1:** General characteristics of the included drug discontinuation trials (*n =* 103)

		no. (%)
Publication year	1976–79	1 (1.0)
	1980–89	21 (20.4)
	1990–99	23 (22.3)
	2000–09	40 (38,8)
	2010–20	18 (17.5)
Region	USA	22 (21.4)
	South America	2 (1.9)
	Africa	2 (1.9)
	Europe	64 (62.1)
	Asia	11 (10.7)
	Oceania	2 (1.9)
Evaluated (discontinued) active substances	Immunosuppressants	19 (18.4)
	Steroids	11 (10.7)
	Antiepileptic drugs	6 (5.8)
	Antidepressants	10 (9,7)
	Antipsychotics	21 (20.4)
	Infection prophylaxis in chronic diseases	6 (5.8)
	Antihypertensive agents	9 (8.7)
	Sedatives and Anxiolytics	5 (4.8)
	Cardiac glycosides	2 (1.9)
	Antiviral agents	2 (1.9)
	Acid suppressive agents	2 (1.9)
	Other drugs for chronic conditions (e.g. Alendronate for the treatment of osteoporosis or Theophylline for the treatment of Chronic Obstructive Pulmonary Disease)	10 (9.7)
Follow up time (Period after the medication has been discontinued/phased out in a study participant)	≤ 6 months	40 (38.8)
	month	18 (17.5)
	> 1 year	18 (17.5)
	Not specified	27 (26.2)
Funding by (*n =* 113) (more than one funding source in 9 studies)	Pharmaceutical industries	14 (12.4)
	Governmental institutions	24 (21.2)
	Voluntary Foundations	19 (16.8)
	Not specified	56 (49.6)

This table summarizes the characteristics of the 103 drug discontinuation trials, including publication year, region, active substances evaluated, follow-up time after discontinuation, and funding sources

### Motivation for treatment and drug discontinuation

Nearly two-thirds (62.1%) of the drugs analyzed were initially prescribed to improve the prognosis of a chronic disease, such as statins for coronary heart disease (see Table 2). Approximately one-third (31.1%) was administered for symptom control. Discontinuation studies involving drugs originally prescribed for curative (2.9%) or prophylactic (4.9%) purposes were comparatively rare.

In nearly 40% of the studies, side effects were the primary reason for investigating drug discontinuation. This was particularly relevant in patients with stable disease, where long-term treatment caused frequent or severe adverse effects. Common examples include antiepileptics, antipsychotics, diuretics, glucocorticoids, and other immunosuppressants.

In more than half of the studies (53.4%) discontinuation was motivated by uncertainty regarding the clinical benefit of the drug or used as an indirect method of evaluating its efficacy (see Table 2). This was especially relevant for antidepressants and antipsychotics. For instance, the DESEP study examined dementia patients receiving long-term antidepressants and monitored depressive symptoms following discontinuation [21].

A smaller proportion of studies (4.9%) investigated drugs that had entered clinical use prior to the implementation of rigorous trial requirements. In these cases, discontinuation served as a means to assess real-world effectiveness—such as in studies on long-term nitrate therapy for angina [22].

In a few cases (2.9%), discontinuation was part of an intentional therapy adjustment, typically after a predefined treatment target had been reached (see Table 2). One example is the RCT by Lee et al. [23], in which statins were discontinued after achieving a target LDL cholesterol level.

**Table 2:** Overview of drugs under study in the included trials (*n =* 103)

		no. (%)
Initial therapeutic objective	Symptom control—alleviation of discomfort, but no effect on the underlying disease (for example: Analgesic for pain syndromes or Metoclopramid or nausea)	32 (31.1)
	Cure—causal treatment of the disease; with the aim of cure (for example: H2-receptor antagonist for duodenal ulcer)	2 (1.9)
	Prognostic improvement—disease modification (for example: ACE inhibitor for heart failure or statin for coronary heart disease)	64 (62.1)
	Prophylaxis—currently non-existent but impending problem is to be prevented (for example: Antifungal drug to prevent mycosis in HIV)	5 (4.9)
Motive for investigating drug discontinuation from the author’s perspective	Side effects of drug	40 (38.8)
	Doubts about the benefit of drug (unclear/low/absent benefit) or indirectly proof of efficacy by discontinuation	55 (53.4)
	Established therapy despite low evidence level	5 (4.9)
	Modified therapy regime in course of time	3 (2.9)

This table summarizes the initial therapeutic objectives and the motivations for investigating drug discontinuation in the 103 included trials

### Primary outcome definitions

In 66% of the studies primary outcomes were explicitly defined. In approximately one-third, the outcome was not directly stated but could be inferred from contextual information such as the sample size calculation. In a small proportion of studies (4.9%), no primary outcome could be discerned (see Table 3).

Most studies used a single primary outcome; composite and co-primary outcomes were relatively uncommon. Clinical endpoints were employed in the majority of trials (59.2%), while subclinical or mixed outcomes were used less frequently. Only a small subset of studies (5%) included discontinuation rates as part of the primary outcome (see Table 3).

The majority of trials (65.0%) focused on outcomes related to the condition for which the drug had originally been prescribed (JT). In these cases, researchers typically monitored symptom deterioration, relapse, or adverse events following discontinuation. Demonstrating that the discontinuation of therapy does not lead to clinical worsening is highly relevant. Thus, such studies are ideally based on a non-inferiority design, including a clearly defined non-inferiority margin and appropriate sample size calculation.

In 20.4% of studies, outcomes were selected to assess the disadvantages of ongoing therapy (JW) such as side effects, costs, or patient burden. Here, superiority hypotheses are generally more appropriate, as they test whether discontinuation leads to measurable improvements compared to continuation.

Both JT and JW challenges were reflected in 10 out of 103 studies. For instance, Curran et al. [24] investigated benzodiazepine withdrawal. Improvements in cognitive function and psychomotor performance supported discontinuation (JW), while outcomes such as mood, sleep, and quality of life reflected the original treatment intent (JT).

Regarding temporal orientation, the majority of primary outcomes (66%) were based on the number of events occurring during follow-up. Fewer studies employed time-to-event or pre-post comparisons (see Table 3).

**Table 3:** Overview of primary outcome definitions and challenges (*n =* 103)

		no. (%)
Presentation of primary outcome(s)	Primary Outcome(s) explicitly defined in article	68 (66.0)
	Primary Outcome(s) not explicitly described, but can be assumed from context or the section on sample size calculation	30 (29.1)
	No primary outcome(s) discernible	5 (4.9)
Structure of primary outcome(s)	Single	69 (67)
	Composite outcome	11 (10.7)
	Co-primary outcome	5 (4.9)
	Multiple outcomes, neither composite nor co-primary	13 (12.6)
	No primary outcome(s) discernible	5 (4.9)
Type of primary outcome(s)	Discontinuation rate	5 (4.9)
	Subclinical—endpoint not noticeable to the patient (e.g. laboratory values, imaging findings)	26 (25.2)
	Clinical – endpoints noticeable for the patient (e.g. symptoms, quality of life, morbidity, death)	61 (59.2)
	Combination of subclinical and clinical endpoint	6 (5.8)
	No primary outcome(s) discernible	5 (4.9)
Challenges	Justification of treatment – Outcome selection focus is on the disease and its course for which the medication was originally prescribed	67 (65.0)
	Justification of withdrawal—Outcome selection focuses on disadvantages of drugs	21 (20.4)
	Both (more than one outcome referring to justification of treatment and withdrawal)	10 (9.7)
	No primary outcome(s) discernible	5 (4.9)
The role of"time"in endpoint definition	Number of events during follow-up time (frequency of events, e.g., number of myocardial infarctions)	68 (66.0)
	Time to event, typically in weeks or month (definition can be clinical, biochemical, imaging etc.)	2 (1.9)
	Difference of a metric endpoint between study start and end (e.g., cognitive function after six weeks of follow-up)	28 (27.2)
	No primary outcome(s) discernible	5 (4.9)

This table summarizes the presentation, type, and challenges of primary outcomes, as well as the role of time in endpoint definition in the included trials

### Hypotheses evaluated

In 43 (41.7%) of the included studies, the hypothesis being investigated was explicitly stated. Specifically, 10 studies described a non-inferiority hypothesis, and 32 studies presented a superiority hypothesis (see Table 4). However, in more than half of the studies (58.3%), the hypothesis type was not explicitly mentioned.

Table 4 shows the cross-classification of hypothesis types as reported by study authors and as assessed by us. We categorized all studies according to the most relevant research question, taking into account the study topic and clinical context. Based on our assessment, a non-inferiority hypothesis would have been appropriate in nearly half of all studies (48.5%).

Among the 32 studies classified as superiority trials by the original authors, 16 (50.0%) were also categorized as superiority trials in our assessment, while 12 (37.5%) were categorized as non-inferiority trials and 4 (12.5%) as addressing both. Of the 10 studies described as non-inferiority trials, 9 (90.0%) were confirmed as such in our assessment, and 1 (10.0%) was assigned to both. Among the 60 studies without a stated hypothesis type, we assessed 29 (48.3%) as non-inferiority trials, 15 (25.0%) as superiority trials, and 16 (26.7%) as addressing both (see Table 4). For additional details, please refer to Supplementary Table 1, which provides further breakdowns and specific data points.

Our findings suggest that several studies might have been more appropriately conceptualized as non-inferiority trials, although they were not presented as such. One example is the study by Chen et al. [25], which examined quetiapine discontinuation in patients with a first remitted psychotic episode. While the authors expected higher relapse rates in the discontinuation group, from a clinical perspective, a non-inferiority design would have been more suitable to assess whether discontinuation leads to recurrence within an acceptable margin, particularly considering the potential side effects of quetiapine.

**Table 4:** Underlying hypotheses: authors'perspective and our interpretation (*n =* 103)

	Appropriate hypotheses according to us			Total (study authors)
Hypotheses according to the study authors	Superiority	Non-inferiority	both	
Superiority	16	12	4	32
Non-inferiority	0	9	1	10
both	0	0	1	1
No specification	15	29	16	60
Total (us)	31	50	22	103

This table compares the hypotheses specified by the study authors (e.g., superiority, non-inferiority) with our interpretation

### Hypotheses and the JW/JT challenges

In our analysis of 67 studies with a primary outcome addressing JT, 41 studies aimed to investigate non-inferiority. In six of these studies, however, a non-inferiority hypothesis was not explicitly stated. Instead, outcomes related to JW were used, although the research question appeared more closely aligned with a non-inferiority framework focused on JT. Concrete examples of such cases are presented and discussed below.

In 13 studies, a JW outcome was investigated, and we considered a superiority design to be appropriate for these. Conversely, 18 studies employed a superiority design while addressing outcomes related to JT. For instance, McMillan et al. [26] used a statistical approach consistent with superiority testing (t-test or Mann–Whitney U test) to examine the discontinuation of growth hormone substitution and its effect on psychological well-being (JT) in patients with severe hormone deficiency. This suggests that their primary motivation was to demonstrate effectiveness, which seems plausible given the state of the evidence.

Overall, we observed two recurring patterns: (1) a shift in the selected outcome type (between JT and JW) and (2) the use of a superiority design where a non-inferiority design may have been more appropriate. In several cases, an outcome switch from JT to JW was observed, which potentially avoided the methodological requirements of a non-inferiority trial, such as a larger sample size.

## Discussion

### Summary of our findings

Our review of discontinuation studies (type 1 according to Viniol et al. [12]) reveals the complexity of this kind of research, especially in selecting the primary outcome and formulating study hypotheses. We explored the motivations, challenges, and consequences of discontinuation studies regarding primary outcome selection.

In most studies, primary outcomes were clearly defined, with a majority using a single primary outcome and some using either composite or co-primary outcomes. Clinically relevant endpoints such as symptoms, quality of life, and death were frequently used, while subclinical measurements were less common. Discontinuation rates were considered in only a small fraction of the studies. However, our evaluation also highlights that discontinuation studies pose substantial methodological challenges—particularly regarding outcome specification, hypothesis formulation, and study design.

### Choice of outcomes and formulation of hypotheses

Discontinuation studies must address two distinct perspectives: the justification for continuing treatment (JT) and for withdrawing it (JW). While JT outcomes assess disease control and typically necessitate non-inferiority designs, JW outcomes highlight drawbacks of ongoing treatment and are best addressed using superiority designs.

Although most studies aligned broadly with these principles, several cases revealed inconsistencies between stated goals and methodological choices. For example, Höcker et al. [27] investigated steroid withdrawal in pediatric kidney transplant recipients and chose longitudinal growth (JW) as the primary endpoint. However, the clinically critical outcome—graft function (JT)—was only analyzed descriptively as a secondary endpoint. A non-inferiority design focused on graft survival would have been more appropriate, though it would have required a substantially larger sample size (see Table 5).

**Table 5:** Study example – steroid discontinuation in pediatric kidney transplantation

Study description	*42 low-immunologic risk pediatric kidney transplant recipients on cyclosporine micro-emulsion, mycophenolate mofetil, and corticosteroids were randomly assigned to either continue steroids or withdraw them over 3 months. The primary outcome was longitudinal growth*
Problem Statement	The key issue with steroid withdrawal is the safety of graft function. Theoretically, safety should be the primary endpoint and adequately powered to show at least non-inferiority of the proposed regimen
Sample Size Considerations	A non-inferiority study would require at least 196 patients per treatment arm to detect a ≤ 5% difference in glomerular filtration rate (GFR) with 90% power and a 15% coefficient of variation. Due to the limited number of pediatric renal transplant patients, such a study would not be feasible
Reference	Höcker B, Weber LT, Feneberg R, Drube J, John U, Fehrenbach H et al. *Nephrol Dial Transplant* 2010; 25(2):617–24. 10.1093/ndt/gfp506.

This table presents a typical study example that illustrates the challenges involved in hypothesis formulation and outcome selection

A similar issue arises in the study by Kendrick et al. [28], which prioritized cognitive function (JW) after antiepileptic withdrawal, while seizure frequency (JT) was a secondary outcome and not part of the sample size calculation. These examples illustrate that, while both perspectives are often acknowledged, they are rarely addressed equally in study design and statistical planning.

This imbalance poses a problem. As shown in the study by Höcker et al. [27], the omission of an adequately powered primary endpoint for a rare but severe outcome—such as graft failure—limits interpretability. Severe outcomes not only require narrow non-inferiority margins but also demand ethical scrutiny, as they cannot be dismissed as rare side effects. Statistical parameters like alpha, beta, and acceptable risk thresholds must therefore be clinically and ethically justified, not treated as formalities.

Borm et al. [29] emphasized that a study's evidential strength should be judged by its overall success, not just by the power of isolated results. They further developed this concept, stressing that the selection of endpoints should not be based solely on statistical properties—clinical relevance must always take precedence [30]. In this light, a trial that shows improved growth (JW) after discontinuing immunosuppressive therapy but cannot rule out an increased risk of graft rejection (JT) remains inconclusive. A more meaningful design would enable conclusions on both dimensions—treatment safety and benefit of withdrawal—even if this increases the complexity and resource demands of the study.

Despite this, none of the 103 studies we reviewed explicitly defined both JT and JW as co-primary endpoints. Most studies chose a single primary outcome, and only five included co-primary outcomes. The likely reason is feasibility: powering a study for both hypotheses often requires much larger sample sizes. Nevertheless, integrating both perspectives is essential to ensure that drug discontinuation studies are methodologically sound and clinically meaningful.

### Clinical relevance

In clinical studies, researchers choose between clinical endpoints and surrogate markers based on the study goals, constraints, and disease characteristics. Clinical endpoints, which assess a patient's well-being, function, or survival, provide the most reliable measure of an intervention's impact but often require long follow-ups or large samples. Surrogate markers are useful in phase 2 trials to assess feasibility but become problematic in definitive phase 3 trials [31].

The validity of a surrogate depends on how well it captures the causal pathways between the intervention and the outcome. For example, Thyroid Stimulating Hormone (TSH) is a justified surrogate when discontinuing thyroid hormones, as regular monitoring helps prevent overt hypothyroidism and its associated harms. In contrast, Low-Density Lipoprotein cholesterol (LDL-C), as used by Lee et al. [23], is an inadequate surrogate for cardiovascular events. While cholesterol levels correlate with cardiovascular disease, they do not reliably predict individual risk [32, 33].

Thus, monitoring TSH to prevent overt hypothyroidism is appropriate when discontinuing thyroid hormones. However, using LDL-C instead of clinical events to assess statin discontinuation in older adults is inappropriate.

### Discontinuation rate and follow-up

Discontinuation rates can be a key outcome in drug discontinuation studies [13]. When a drug is stopped as part of the study protocol, reinstitution rates can indicate"failed discontinuation"and offer valuable insights. Disease flares or symptom recurrence are common reasons for resuming treatment, reflecting JT. For example, restarting antipsychotics due to neuropsychiatric symptoms or reintroducing thyroxine when TSH levels rise are both instances of JT. In these cases, TSH could also serve as a safety measure.

However, in patients who tolerate increased disease activity after discontinuation, a mechanism justifying withdrawal can be identified. For instance, in the treatment of autoimmune diseases, glucocorticoids are often discontinued due to metabolic side effects, such as weight gain [34].

Psychological factors, like patient or clinician reactions, may also influence the decision to reinstitute treatment, particularly in blinded trials. For example, statin discontinuation could reveal unmasked treatment arms due to rising LDL-cholesterol levels, leading to concerns and, consequently, treatment resumption.

Thus, the discontinuation rate is a complex measure that reflects JT, JW, and psychological factors. Additional outcomes may be needed to assess the reasons behind treatment reinstitution, depending on the drug and its indication. The follow-up period must also be considered when evaluating discontinuation rates. For diseases with long-term clinical consequences, such as statin use in cardiovascular prevention, longer follow-up is necessary to capture adverse events, like stroke. If a strong surrogate marker exists, such as HBV DNA levels after discontinuing tenofovir, a shorter follow-up period may be justified [35].

### What do others say?

In 2022, the US Deprescribing Research Network (USDeN) published recommendations on outcome selection in discontinuation studies [36]. A year later, Nizet et al. compared their systematic review of deprescribing implementation trials with these recommendations [37]. Unlike our approach, they did not distinguish between the three types of discontinuation studies [12] and instead analyzed studies of all three types together. In contrast, we focused specifically on Type 1 studies. Given the differing goals and design requirements, we advocate for considering these study types separately. Additionally, the authors did not address the key challenge of discontinuation studies—balancing JT and JW—and its implications for study design and hypothesis formulation. However, they underscored the importance of clinically relevant outcomes, adequate power, and sufficient follow-up time.

### Strength and limitations of our review

This scoping review provides a comprehensive analysis of studies on drug discontinuation, examining motivations, challenges, outcomes, and hypotheses. Based on an extensive literature search, it also proposes a systematic framework to categorize key research questions and guide future studies.

Our review specifically focused on randomized controlled trials (RCTs) of type 1 drug discontinuation studies. Although we may have overlooked relevant findings from other study types, we believe this limitation is justified given the higher risk of bias associated with non-RCT designs. Therefore, we chose to focus solely on RCTs to ensure the reliability of our analysis.

The studies included in our review were limited to those examined by Grede et al., who published a scoping review in 2023 covering publications up to 2020. Since our study aims to develop a structured synthesis rather than provide a fully up-to-date systematic review of the drug discontinuation literature, we do not believe extending the time frame would have substantially altered our findings. While the field of drug discontinuation is evolving rapidly, the 103 studies included in our analysis provide a solid and comprehensive foundation for this work. The primary objective of this study is to synthesize and conceptualize existing knowledge, and the patterns, barriers, and facilitators of drug withdrawal identified in these studies form the basis of our model.

Although two authors independently interpreted the hypotheses, some subjectivity inevitably remains in the interpretation process. This subjectivity may have influenced of our conclusions regarding study designs and hypotheses to some extent.

## Conclusion: recommendations of outcome selection in drug discontinuation studies

In conclusion, drug discontinuation studies must address both key clinical and methodological challenges. The selection of endpoints should prioritize clinical relevance, emphasizing meaningful outcomes or appropriate surrogate markers over purely statistical considerations. This approach should inform the formulation of hypotheses, carefully balancing non-inferiority and superiority designs. Such studies typically require large sample sizes and extended follow-up periods.

The field of drug discontinuation research is rapidly evolving, with many questions still to be answered. These include the comparative valuation of outcomes, the definition of error margins, and strategies for determining sample sizes. Securing funding remains a significant challenge due to the lack of commercial incentives, and public or independent funding sources are often insufficient to support the scale of these studies. Governments must play a central role in addressing this funding gap by supporting research that aligns with public health priorities.

The example of Canada, with initiatives like the Canadian Institutes of Health Research (CIHR) and the Canadian Deprescribing Network (CaDeN), highlights how national networks can drive research on drug discontinuation and the development of specific guidelines. Similar efforts, along with international collaboration, are essential to advancing evidence-based approaches to medication discontinuation and ensuring the safe reduction of unnecessary or potentially harmful drugs [38].

## Supplementary Information

Supplementary Material 1: Characteristics and References of Included Trials [39–122]

## Data Availability

The datasets used and/or analysed during the current study are available from the corresponding author on reasonable request.

## Abbreviations

ACE: Angiotensin-Converting Enzyme
AED: Antiepileptic Drugs
CIHR: Canadian Institutes of Health Research
DNA: Deoxyribonucleic Acid
GFR: Glomerular Filtration Rate
HBV: Hepatitis B Virus
HIV: Human Immunodeficiency Virus
JT: Justification of Treatment
JW: Justification of Withdrawal
LDL-C: Low-Density Lipoprotein Cholesterol
PPI: Proton Pump Inhibitor
PRISMA: Preferred Reporting Items for Systematic Reviews and Meta-Analyses
RCT: Randomized Controlled Trial
SPSS: Statistical Package for the Social Sciences
TNF: Tumor Necrosis Factor
TSH: Thyroid-Stimulating Hormone
US: United States
USA: United States of America
USDeN: US Deprescribing Research Network
