# Supplementary material for: Trials evaluating drug discontinuation: a scoping review sub-analysis focusing on outcomes and research questions
Source: BMC Med Res Methodol. 2025 May 27;25:146. doi: 10.1186/s12874-025-02597-z (PMC12108048; doi:10.1186/s12874-025-02597-z)
Supplement: Supplementary file 1 — Supplementary Material 1: Characteristics and References of Included Trials [39–122] [file 12874_2025_2597_MOESM1_ESM.docx]

# Characteristics and References of Included Trials

| ***No.*** | ***Authors, year, Reference*** | ***Primary Endpoint verbatim*** | ***Discontinued drug (drug class)*** | ***Underlyinig disease*** | ***Original therapeutic objective (cure, symptom control, prognostic improvement, prophylaxis)*** | ***Motive for investigating drug discontinuation (Side effects, proof of efficacy, changed therapy regime, Unclear/little/no benefit)*** | ***Setting*** | ***Sample size*** | ***Follow-up duration*** | ***Structure (one primary outcome, composite, co-primary, multiple) / type of primary outcomes (clinical, subclinical, both, discontinuation rate)*** | ***Hypotheses according to authors*** | ***Hypotheses according to us*** | ***Challenges: JT/JW/both*** | ***Funding*** |
| --- | --- | --- | --- | --- | --- | --- | --- | --- | --- | --- | --- | --- | --- | --- |
| *1* | *Abramowicz et al. 2012 (39)* | Creatinine clearance at 6 months after complete withdrawal | Cyclosporin A  (Immunosuppressants) | Stable kidney transplant recipients | Prognostic improvement | Side effects | Outpatient | 187 | 3-6 months | One primary endpoint/subclinical | Superiority | Non-inferiority | JW | Pharmaceutical industries |
| *2* | *Andrews et al. 1976 (40)* | Relapse | Phenotiazine (Antipsychotics) | Acute schizophrenia | Prognostic improvement | Proof of effectiveness | Inpatient | 31 | 9-12 months | One primary endpoint/clinical | No specification | Non-inferiority | JT | Governmental institutions |
| *3* | *Baker et al. 1983 (41)* | No primary outcome discernible | Neuroleptic medication (fluphenazine enanthate, trifluoperazine, thioridazine, fluphenazine decanoate, chlorpromazine, pimozide, perphenazine, mesoridazine, haloperidol, loxapine Benztropine mesylate  (Antipsychotics) | Chronic schizophrenic | Symptom control | Side effects | Not specified | 28 | < 2-3 years | No primary endpoint discernible | No specification | Combined | No specification | Not specified |
| *4* | *Ballard et al. 2008 (42)* | total SIB score for evaluation of global cognitive functioning (change from baseline to 6 month) | Thioridazine, Chlorpromazine, Haloperidol, Trifluoperazine or Risperidone (Antipsychotics) | Behavioural or psychiatric disturbance in dementia | Prognostic improvement | Side effects | Inpatient | 165 | Not specified | One primary endpoint/clinical | Superiority | Combined | JT | Voluntary foundations |
| *5* | *Ballard et al. 2004* | behavioral symptoms using the Neuropsychiatric Inventory (NPI) | Thioridazine, Chlorpromazine, Haloperidol, Trifluoperazine, Risperidone  (Antipsychotics) | Dementia | Symptom control | Unclear/little/no benefit | Inpatient | 100 | 1-2 months | One primary endpoint/clinical | No specification | Non-inferiority | JT | Voluntary foundations |
| *6* | *Ballard et al. 2015* | Function (Bristol Activities of Daily Living Scale (BADLS)) and agitation (Cohen-Mansfield Agitation Inventory (CMAI) | Memantine (other drugs for chronic conditions) | Alzheimer | Symptom control | Proof of effectiveness | Not specified | 199 | <1-2 years | Composite/clinical | Noninferiority | Combined | JT | Pharmaceutical industries |
| *7* | *Barton et al. 1991* | seizure recurrence | Phenobarbitone, Phenytoin, Carbamazepine, Valproate, Primidone, Ethosuximide (Antiepileptic drugs) | Epilepsy | Symptom control | Side effects | Not specified | 1013 | 1-2 months | One primary endpoint/clinical | No specification | Noninferiority | JT | Not specified |
| *8* | *Bergh et al. 2012* | Score differences between study groups in the Cornell scale of depression in dementia and the neuropsychiatric inventory (10 item version) after 25 weeks | Escitalopram, Citalopram, Sertraline or Paroxetine (Antidepressants) | Alzheimer’s disease, dementia or vascular dementia, and neuropsychiatric symptoms (but no depressive disorder) | Prognostic improvement | Proof of effectiveness | Inpatient | 128 | Not specified | Coprimary/clinical | Superiority | Noninferiority | JT | Governmental institutions |
| *9* | *Bialos et al. 1982* | Appearance of depressive episode | Amitryptiline  (Antidepressants) | Depression | Symptom control | Side effects | Outpatient | 17 | 3-6 month | One primary endpoint/clinical | Superiority | Superiority | JT | Not specified |
| *10* | *Branchey et al. 1981* | Relapse (level of psychopathology) and symptoms of tardive dyskinesia | Not specified; all neuroleptics were replaced by equivalent doses of Loxapine hydrochloride in active form  (Antipsychotics) | Schizophrenia | Prognostic improvement | Side effects | Inpatient | 33 | Not specified | Multiple/clinical | No specification | Combined | Both | Not specified |
| *11* | *Buti et al. 2015 (35)* | sustained response | Tenofovir  (Antiviral agents) | Hepatitis B | Prognostic improvement | Changed therapy regimen | Outpatient | 8 | 3-6 months | Composite/subclinical | No specification | Noninferiority | JT | Governmental institution |
| *12* | *Bykerk et al 1991 (43)* | Time to the development of a clinical flare-up of SLE on the basis of the development of specific clinical manifestations (defined according American Rheumatism Association Glossary) or an increase in their severity | Hydroxychloroquine (Immunosuppressants) | Systemic lupus erythematosus | Prognostic improvement | Proof of effectiveness | Outpatient | 47 | Not specified | Co-primary/clinical | Superiority | Superiority | JT | Voluntary foundations |
| *13* | *Campbell et al. 1999 (44)* | falls | Benzodiazepine, any other hypnotic or any antidepressant or major tranquilizer, psychotropic medication  (Sedatives and anxiolytics) | Not specified | Prognostic improvement | Side effects | Outpatient | 93 | 3-6 months | One primary endpoint/clinical | Superiority | Superiority | JW | Not specified |
| *14* | *Campbell et al. 2012 (45)* | Episodes of malaria and diarrhea | Cotrimoxazole  (Infection prophylaxis in chronic disease) | HIV | Prophylaxis | Unclear/little/no benefit | Inpatient | 836 | < 1-2 years | Coprimary/clinical | No specification | Noninferiority | JT | Governmental institution,  Voluntary foundations |
| *15* | *Chaiwarith et al. 2013 (46)* | incidence of patients who died or developed any of the following Ois (opportunistic infections): PCP, cerebral toxoplamosis, disseminated MAC infection, disseminated penicillinosis merneffei, disseminated histoplasmosis and cryptococcal meningitis | Cotrimoxazol, antifungal prophylaxis  (Infection prophylaxis in chronic disease) | HIV | Prognostic improvement | Side effects | Outpatient | 74 | 3-6 months | Composite/clinical | Noninferiority | Noninferiority | JT | Pharmaceutical industries |
| *16* | *Chen et al. 2010 (47)* | Relapse | Quetiapine  (Antipsychotics) | First episode psychosis | Symptom control | Proof of effectiveness | Outpatient | 178 | Not specified | One primary endpoint/clinical | Superiority | Superiority | JT | Pharmaceutical industries,  Governmental institution |
| *17* | *Choudhury et al. 2007 (48)* | COPD exacerbation frequency  (unreported, moderate, severe) | Inhaled corticosteroids (Steroids) | COPD | Symptom control | Proof of effectiveness | Outpatient | 260 | 3-6 months | One primary endpoint/clinical | Superiority | Combined | JT | Pharmaceutical industries,  Governmental institution,  Voluntary foundations |
| *18* | *Cook et al. 1986 (49)* | Evaluation of the relapse rate | Tricyclic antidrepressant: Desipramine, Amitriptyline, Doxepin, Imipramine (Antidepressants) | Depression | Symptom control | Proof of effectiveness | Outpatient | 15 | 9-12 months | One primary endpoint/clinical | Superiority | Noninferiority | JT | Not specified |
| *19* | *Coulter et al. 1988 (50)* | Seizure frequency | Barbiturate (Phenobarbital, Primidone)  (Antiepileptic drugs) | Epilepsy | Prognostic improvement | Proof of effectiveness | Inpatient | 25 | Not specified | One primary endpoint/clinical | Superiority | Noninferiority | JT | Not specified |
| *20* | *Curran et al. 2003 (24)* | Changes in patients’ cognitive function, quality of life, mood and sleep | Benzodiazepine (Sedatives and Anxiolytics) | Long-term Benzodiazepine users | Symptom control | Side effects | Outpatient | 192 | 9-12 months | Multiple/clinical | No specification | Combined | Both | Governmental institution |
| *21* | *De Jonge et al. 1994 (51)* | Change in volumetrically determined ankle oedema | Diuretic drugs (Furosemide, Triamterene, Amiloride, Hydrochlorothiazide, Epitizide, Bumetanide, Chlortalidone, Mefruside, Spironolactone)  (Antihypertensive agents) | Ankle odema | Prognostic improvement | No evidence | Outpatient | 63 | Not specified | One primary endpoint/clinical | Superiority | Noninferiority | JT | Not specified |
| *22* | *Farmer et al. 2006 (52)* | Incidence of biopsy proven acute cellular rejection 1 year following steroid withdrawal | Prednisone  (Steroids) | Renal transplantation | Symptom control | Side effects | Outpatient | 92 | < 1-2 years | One primary endpoint/subclinical | Superiority | Combined | JT | Not specified |
| *23* | *Findlay et al. 1989 (53)* | No primary outcome discernible | Thioridazine  (Antipsychotics) | Senile dementia, Alzheimer type | Prognostic improvement | Proof of effectiveness | Inpatient | 36 | Not specified | Missing | No specification | Combined | Missing | Not specified |
| *24* | *George et al. 2003 (54)* | Reccurence of anginal symptoms | Nitrates (Antihypertensive agents) | Heart failure | Symptom control | Unclear/little/no benefit | Outpatient | 120 | Not specified | One primary endpoint/clinical | No specification | Noninferiority | JT | Not specified |
| *25* | *Ghaemi et al. 2010 (55)* | Mean change on the depressive subscale of the CMF (STEP-BD Clinical Monitoring Form) evaluation of the less studied subsyndromal component | Bupropion, Venlafaxine, Paroxetine, Citalopram  (Antidepressants) | Bipolar depression | Symptom control | Unclear/little/no benefit | Outpatient | 70 | < 3-4 years | One primary endpoint/clinical | Superiority | Superiority | JT | Not specified |
| *26* | *Giller et al. 1985 (56)* | Completion of six-month protocol or the appearance of a depressive episode | Amitriptyline (Antidepressants) | Depression | Prognostic improvement | Proof of effectiveness | Outpatient | 19 | 3-6 months | One primary endpoint/clinical | No specification | Noninferiority | JT | Not specified |
| *27* | *Gleissner et al. 2006 (57)* | Creatinine serum levels / calculated creatinine clearance (cGFR) after 6 months as compared to baseline levels | Cyclosporine (Immunosuppressants) | Heart Transplant Patients with renal failure | Prognostic improvement | Side effects | Not specified | 39 | 9-12 months | One primary endpoint/subclinical | No specification | Noninferiority | JW | Pharmaceutical industries |
| *28* | *Gøtzsche et al. 1996 (58)* | Treatment failure and patient’s well-being on a 5-point scale, number of tender joints on palpation judged by the physician and number of painful joints judged by the patient | Methotrexate, Penicillamine or Sulfasalazine  (Immunosuppressants) | Rheumatoid arthritis | Prognostic improvement | Side effects | Outpatient | 112 | Not specified | Composite/clinical | Noninferiority | Noninferiority | JT | Voluntary foundations |
| *29* | *Greil et al. 1984 (59)* | Symptoms of tardive dyskinesia | Anticholinergics (Benperidol, Thioridazine, Fluphenazine, Levomepromazine, Perphenazine, Fiupenthixo, Haloperidol)  (Antipsychotics) | Chronic schizophrenic | Prognostic improvement | Side effects | Not specified | 10 | > 5 years | One primary endpoint/clinical | No specification | Superiority | JW | Not specified |
| *30* | *Habraken et al. 1997 (60)* | The level of daily functioning according to the Geriatrics Behavior Observation Scale | Lorazepam (Sedative and anxiolytic) | Sleep disorders | Prognostic improvement | Unclear/little/no benefit | Inpatient | 55 | 3-6 months | One primary endpoint/clinical | No specification | Combined | JW | Governmental institution |
| *31* | *Hardy et al. 1997 (16)* | Depression recurrence | Lithium (Antidepressants) | Unipolar depression | Symptom control | Proof of effectiveness | Outpatient | 12 | < 4-5 years | One primary endpoint/clinical | No specification | Noninferiority | JT | Governmental institution |
| *32* | *Hausberg et al. 2006 (61)* | Muscle sympathetic nerve activity (MSNA) | Cyclosporine (Immunosuppressants) | Chronic allograft nephropathy | Prognostic improvement | Side effects | Outpatient | 24 | Not specified | One primary endpoint/surrogate | Superiority | Superiority | JW | Not specified |
| *33* | *Hawthorne et al. 1992 (62)* | Rate of relapse | Azathioprine (Immunosuppressants) | Ulcerative colitis | Prognostic improvement | Side effects | Outpatient | 67 | Not specified | One primary endpoint/clinical | Superiority | Superiority | JT | Not specified |
| *34* | *Hessen et al. 2006 (17)* | Effect of discontinuation of AEDs in patients receiving monotherapy on measures of attention, reaction time, and speed of information processing | Carbamazepine, Valproate, Phenytoin, Phenobarbital, Lamotrigine  (Antiepileptic drugs) | Epilepsy | Prognostic improvement | Side effects | Outpatient | 150 | Not specified | Multiple/clinical | No specification | Superiority | JW | Not specified |
| *35* | *Hessen et al. 2007 (63)* | Behavioral complaints evaluated with the Norwegian edition of the MMPI-2 | Carbamazepine, Valproate, Phenytoin, Phenobarbital, Lamotrigine (Antiepileptic drugs) | Epilepsy | Prognostic improvement | Side effects | Outpatient | 150 | Not specified | One primary endpoint/clinical | Superiority | Superiority | JW | Not specified |
| *36* | *Höcker et al. 2009 (27)* | standardized longitudinal growth that is change in height SDS | Prednisone (Steroids) | Pediatric renal transplantation | Prognostic improvement | Side effects | Not specified | 42 | 3-6 months | One primary endpoint/subclinical | Superiority | Noninferiority | JW | Pharmaceutical industries |
| *37* | *Hollander et al. 1997 (64)* | Percentage of successful prednisone withdrawal | Prednisone (Steroids) | Kidney Transplant Patients | Prognostic improvement | Side effects | Not specified | 84 | 7-9 months | One primary endpoint/ discontinuation rate | No specification | Combined | Both | Pharmaceutical industries |
| *38* | *Jellinek et al. 1981 (65)* | No primary outcome discernible | Trihexyphenidyl, Benztropine or Biperiden (Antipsychotics) | Schizophrenia | Prognostic improvement | Proof of effectiveness | Outpatient | 24 | >1-2 years | Missing | No specification | Noninferiority | Missing | Not specified |
| *39* | *Kendrick et al. 1993 (28)* | Mood | Phenytoin, Carbamazepine or Sodium valproate (Antiepileptic drugs) | Epilepsy | Prognostic improvement | Proof of effectiveness | Not specified | 80 | > 3-4 years | One primary endpoint/clinical | No specification | Noninferiority | JW | Not specified |
| *40* | *Kim et al. 2014 (66)* | Proportion of patients with persistently undetectable HBV DNA at weeks 48 and 96 | Lamivudine (LVD) (Antiviral agents) | Chronic hepatitis B (CHB) | Prognostic improvement | Side effects | Outpatient | 72 | 7-9 months | One primary endpoint/subclinical | Noninferiority | Noninferiority | JT | Pharmaceutical industries,  Governmental institutions |
| *41* | *Kirsten et al. 1993 (67)* | Effect on lung function and other physiologic parameters as well as exercise performance, symptoms, overall dyspnea, and quality of life | Theophylline (other drugs for chronic conditions) | COPD | Prognostic improvement | Proof of effectiveness | Not specified | 39 | 3-6 months | Multiple/both | No specification | Superiority | JT | Not specified |
| *42* | *Korman et. al 1980 (68)* | Relapse rate | Cimetidine (Acid suppressive agents) | Duodenal ulcer | Cure | Side effects | Outpatient | 41 | 3-6 months | One primary endpoint/clinical | No specification | Noninferiority | JT | Not specified |
| *43* | *Kosch et al. 2003 (69)* | Arterial compliance and endothelium-dependent flow-mediated vasodilatation | Cyclosporin A (Immunosuppressants) | Renal transplant patients | Prognostic improvement | Side effects | Outpatient | 24 | 3-6 months | Coprimary/ subclinical | No specification | Superiority | JW | Not specified |
| *44* | *Le Cesne et al. 2010 (70)* | Progression-free survival | Imatinib (Immunosuppressants) | Advanced gastrointestinal stromal tumours | Prognostic improvement | Proof of effectiveness | Outpatient | 434 | > 2-3 years | One primary endpoint/subclinical | Noninferiority | Noninferiority | JT | Pharmaceutical industries,  Voluntary foundations |
| *45* | *Lee et al. 2014 (23)* | Rate of relapse of dyslipidemia | Rosuvastatin (other drugs for chronic conditions) | Dyslipidemia | Prognostic improvement | Changed therapy regimen | Outpatient | 99 | <1 month | One primary endpoint/subclinical | Superiority | Superiority | JT | Not specified |
| *46* | *Legendre et al. 2007 (71)* | Change in Glomerular filtration rate (GFR) | Cyclosporine A (Immunosuppressants) | Renal transplant recipients | Prognostic improvement | Side effects | Not specified | 430 | 3-6 months | One primary endpoint/subclinical | No specification | Noninferiority | JT | Pharmaceutical industries |
| *47* | *Lémann et al. 2005 (72)* | Clinical relapse over the 18-month period | Azathioprine (Immunosuppressants) | Crohn’s disease | Symptom control | Proof of effectiveness | Outpatient | 83 | Not specified | One primary endpoint/clinical | Noninferiority | Noninferiority | JT | Voluntary foundations |
| *48* | *Lemos et al. 2014 (22)* | Change in HRQoL scores | Nitrate (Antihypertensive agents) | Stable angina | Prognostic improvement | No evidence | Outpatient | 105 | < 1 months | One primary endpoint/clinical | No specification | Superiority | JW | Governmental institutions |
| *49* | *Lin et al. 2014* | Occurrence of resuming medication | Dutasteride, Doxazosin (other drugs for chronic conditions) | Benign prostatic hyperplasia (BPH) | Symptom control | Unclear/little/no benefit | Outpatient | 117 | 3-6 months | One primary endpoint/discontinuation rate | Noninferiority | Noninferiority | Both | Not specified |
| *50* | *Lossius et al. 2008 (73)* | Seizure relapse and changes in cognitive function at inclusion and after 7 Mo after intervention | Carbamazepine, Valproate, Phenytoin, Phenobarbital, Lamotrigine (Antiepileptic drugs) | Recent onset epilepsy | Symptom control | Side effects | Outpatient | 160 | 3-6 months | Coprimary/clinical | No specification | Combined | Both | Voluntary foundations |
| *51* | *Maland et al. 1983 (74)* | Changes in blood pressure | Thiazide (Chlorthalidone, Hydrochlorothiazide, Triamterene) (Antihypertensives) | Hypertension | Symptom control | Unclear/little/no benefit | Outpatient | 62 | 9-12 month | One primary endpoint/subclinical | No specification | Noninferiority | JT | Not specified |
| *52* | *Margo et al. 1982 (75)* | Relapse | Lithium (Antidepressants) | Bipolar manic depressiveillness, cyclothymic personality | Prognostic improvement | Proof of effectiveness | Inpatient | 12 | 3-6 months | One primary endpoint/clinical | No specification | Noninferiority | JT | Not specified |
| *53* | *Mayur et al. 2000 (76)* | differential therapeutic and side effect responses | Imipramine (Antidepressants) | Depression | Prognostic improvement | Proof of effectiveness | Not specified | 30 | Not specified | Coprimary/clinical | No specification | Combined | Both | Not specified |
| *54* | *McDiarmid et al. 1995 (77)* | Incidences of acute, chronic, or steroid-resistant rejection | Prednisone (Steroids) | Orthotopic liver transplantant recipients | Prognostic improvement | Unclear/little/no benefit | Not specified | 64 | <1 months | One primary endpoint/subclinical | No specification | Combined | JT | Not specified |
| *55* | *McInnis et al. 1985 (78)* | withdrawal syndrome (anxiety, hypotension, tachycardia, physical complaints) and psychotic/psychiatric symptoms, extrapyramidal side effects | Trihexyphenidyl (Antipsychotics) | Extrapyramidal symptoms, schizophrenia | Prognostic improvement | Proof of effectiveness | Inpatient | 22 | 7-9 months | Multiple/clinical | No specification | Superiority | JT | Not specified |
| *56* | *McLennan et al. 1992 (79)* | Prolactin levels in blood | Thioridazine (Antipsychotics) | Senile dementia | Prognostic improvement | Side effects | Inpatient | 66 | Not specified | One primary endpoint/subclinical | No specification | Superiority | JW | Not specified |
| *57* | *McMillan et al. 2003 (26)* | Effect of 3 months’ GH withdrawal on quality of life (QoL) and aspects of health status and psychological well-being | Growth Hormone replacement (other dugs for chronic conditions) | Growth hormone deficiency | Symptom control | Proof of effectiveness | Outpatient | 21 | <1 months | Multiple/clinical | Superiority | Superiority | JT | Not specified |
| *58* | *Mellman et al. 1986 (80)* | Changes in anxiety and plasma cortisol levels, changes in vital signs and sleep | Alprazolam  (Sedative and anxiolytic) | General anxiety, panic disorders | Symptom control | Proof of effectiveness | Not specified | 10 | Not specified | Multiple/both | No specification | Noninferiority | JT | Not specified |
| *59* | *Michalska et al. 2006 (81)* | Difference in vertebral BMD change | Alendronate (other drugs for the treatment of chronic conditions) | Postmenopausal osteoporosis | Prognostic improvement | Side effects | Outpatient | 99 | >4-5 years | One primary endpoint/subclinical | No specification | Noninferiority | JT | Not specified |
| *60* | *Miro et al. 2006 (18)* | Occurrence of TE (toxoplasmic encephalitis) | Toxoplasma gondii Prophylaxis: Sulfadiazine plus pyrimethamine plus folinic acid,  Clindamycin plus pyrimethamine plus folinic acid, other antitoxoplasma therapies (Infection prophylaxis in chronic disease) | Toxoplasmic encephalitis in human immunodeficiency virus (HIV)–infected patients | Prophylaxis | Unclear/little/no benefit | Outpatient | 381 | <1 months | One primary endpoint/clinical | Noninferiority | Noninferiority | JT | Governmental institutions |
| *61* | *Mussini C. et al. 2003 (82)* | confirmed or presumptive diagnosis of PCP or death related to this opportunistic infection | Trimethoprim-Sulfamethoxazole, aerosolized Pentamidine, Atovaquone, Dapsone alone or Dapsone plus Pyrimethamine (Infection prophylaxis in chronic diseases) | HIV | Prophylaxis | Proof of effectiveness | Not specified | 146 | >2-3 years | Composite/both | Superiority | Superiority | JT | Governmental institutions |
| *62* | *Mussini et al. 2000 (83)* | Definitive diagnosis of P. carinii pneumonia, a definitive or presumptive diagnosis of toxoplasmic encephalitis and death related to these opportunistic infections | Trimethoprim/Sulfamethoxazole, aerosolized Pentamidine, Dapsone, Pyrimethamine  (Infection prophylaxis in chronic diseases) | HIV | Symptom control | Proof of effectiveness | Not specified | 708 | >1-2 years | Composite/both | Superiority | Superiority | JT | Governmental institutions |
| *63* | *Odejide et al. 1982 (84)* | Relapse rate | Fluphenazine decanoate (Antipsychotics) | Schizophrenia | Prognostic improvement | Proof of effectiveness | Not specified | 27 | Not specified | One primary endpoint/clinical | No specification | Superiority | JT | Not specified |
| *64* | *Ory-Magne et al. 2014 (85)* | Change from baseline in a Unified Parkinson’s Disease Rating Scale (UPDRS) dyskinesia subscore (items 32 [duration] 1 33 [severity]) | Amantadine (other drugs for chronic conditions) | Parkinson disease | Prognostic improvement | Side effects | Not specified | 57 | Not specified | One primary endpoint/clinical | Superiority | Superiority | JT | Governmental institutions,  Voluntary foundations |
| *65* | *Packer et al. 1993 (86)* | (1) rates of withdrawal from the study due to worsening heart failure, (2) time to withdrawal, and (3) changes in exercise tolerance (as assessed by both time and distance) | Digoxin (Cardiac glycosides) | Chronic heart failure | Prognostic improvement | No evidence | Outpatient | 85 | >1-2 years | Multiple/clinical | No specification | Superiority | JT | Pharmaceutical industries |
| *66* | *Pato et al. 1988 (87)* | recurrence of obsessive-compulsive symptoms  (relapse) | Clomipramin (Antidepressant) | Obsessive-compulsive disorder | Symptom control | Proof of effectiveness | Outpatient | 21 | 9-12 months | One primary endpoint/clinical | No specification | Noninferiority | JT | Not specified |
| *67* | *Petursson et al. 1983 (88)* | Digit Symbol Substitution Test (DSST), Symbol Copying Test (SCT), Cancellation Task (CT), Auditory Reaction Time (RT) and Key Tapping Rate (KTR) | Benzodiazepine (Diazepam, Lorazepam, Oxazepam, Triazolam)  (Sedative and anxiolytic) | Anxiety neurosis, depression, personality disorder with anxiety | Symptom control | Side effects | Outpatient, Inpatient | 22 | 9-12 month | Multiple/clinical | No specification | Superiority | JW | Not specified |
| *68* | *Pincus et al. 2009 (89)* | (study) withdrawal due to patient-reported lack of efficacy versus continuation in the trial for 24 weeks | Prednisone (Steroids) | Rheumatoid arthritis (RA) | Prognostic improvement | Proof of effectiveness | Outpatient | 31 | Not specified | One primary endpoint/clinical | No specification | Noninferiority | JT | Voluntary foundations |
| *69* | *Ratcliffe et al. 1996 (90)* | Changes in plasma creatinine | Prednisone (Steroids) | Renal transplantation | Prognostic improvement | Unclear/little/no benefit | Not specified | 100 | 1-2 months | One primary endpoint/subclinical | No specification | Combined | JT | Not specified |
| *70* | *Reimer et al. 2010 (20)* | Difference in proportion with treatment success after 7 days of therapy between the PPI and the placebo group | PPI (Acid suppressive agents) | Functional dyspepsia | Prognostic improvement | Proof of effectiveness | Outpatient | 78 | 9-12 months | One primary endpoint/discontinuation rate | No specification | Superiority | JW | Governmental institutions |
| *71* | *Rice et al. 2000 (91)* | Average number of COPD exacerbations over a 6-mo study period | Prednisone (Steroids) | COPD | Symptom control | Unclear/little/no benefit | Outpatient | 38 | Not specified | One primary endpoint/clinical | Superiority | Superiority | JT | Not specified |
| *72* | *Ruskin et al. 1991 (92)* | Relapse | Neuroleptics, keine genaue Angabe  (Antipsychotics) | Schizophrenia | Prognostic improvement | Proof of effectiveness | Outpatient | 35 | Not specified | One primary endpoint/clinical | No specification | Noninferiority | JT | Governmental institutions |
| *73* | *Ruths et al. 2008 (93)* | Successful antipsychotic discontinuation | Haloperidol, Risperidone or Olanzapine (Antipsychotics) | Behavioural and psychological symptoms of dementia | Prognostic improvement | No evidence | Inpatient | 55 | Not specified | One primary endpoint/discontinuation rate | No specification | Combined | JW | Not specified |
| *74* | *Ruths et al. 2004 (94)* | Means of behavioral ratings and actigraphy | Haloperidol, Risperidone or Olanzapine (Antipsychotics) | Dementia | Prognostic improvement | Proof of effectiveness | Inpatient | 30 | 1-2 months | Composite/clinical | No specification | Combined | JT | Not specified |
| *75* | *Saksa et al. 2004 (95)* | Recurrence (maniac or depression symptoms) | Perphenezine, Haloperidol, Perphenezine, Thioridazine, Haloperidol, Thiothixene, Risperidone (Antipsychotics) | Bipolar patients | Prophylaxis | Unclear/little/no benefit | Outpatient | 15 | 9-12 months | One primary endpoint/clinical | No specification | Noninferiority | JT | Voluntary foundations |
| *76* | *Sampath et al. 1992 (96)* | Relapse rate | Fluphenazine decanoate (Antipsychotics) | Schizophrenia | Prognostic improvement | Proof of effectiveness | Inpatient | 24 | Not specified | One primary endpoint/clinical | No specification | Noninferiority | JT | Governmental institutions |
| *77* | *Schnuelle et al. 2002 (97)* | Kidney function at 1 year | Mycophenolate-Mofetil or Cyclosporine (Immunosuppressants) | Stable renal transplant recipients | Prognostic improvement | Side effects | Outpatient | 84 | 3-6 months | One primary endpoint/subclinical | Superiority | Noninferiority | JW | Not specified |
| *78* | *Smak et al. 2002 (98)* | First biopsy-proven acute or chronic rejection between 6 and 24 months after transplantation | Prednisone or Cyclosporine (Immunosuppressants) | Renal transplant recipients | Prognostic improvement | Side effects | Outpatient | 212 | 9-12 months | One primary endpoint/subclinical | No specification | Combined | JT | Pharmaceutical industries |
| *79* | *Srivanichakorn et al. 2015 (19)* | Evaluation of glycemic control (HbA1c%) | Sulfonylurea (other drugs for chronic conditions) | Type 2 diabetes | Prognostic improvement | Changed therapy regimen | Outpatient | 32 | 1-2 months | One primary endpoint/ subclinical | Superiority | Superiority | JT | Governmental institutions |
| *80* | *Stellon et al. 1988 (99)* | Adverse effects in term of histological appearance or liver function | Prednisone (Steroids) | Autoimmune chronic active hepatitis | Prognostic improvement | Side effects | Not specified | 47 | 1-2 months | Composite /subclinical | No specification | Noninferiority | JT | Not specified |
| *81* | *Stellon et al. 1985 (100)* | Relapse (biochemical and histological) | Azathioprine (Immunosuppressants) | Autoimmune chronic active hepatitis | Prognostic improvement | Proof of effectiveness | Not specified | 50 | 9-12 months | One primary endpoint/ subclinical | No specification | Superiority | JT | Not specified |
| *82* | *Takata et al. 1992 (101)* | Normotensive diastolic blood pressure | Non-thiazide diuretics, angiotensin-converting enzyme inhibitors (Antihypertensive agents) | Essential hypertension | Prognostic improvement | Side effects | Outpatient | 113 | 3-6 months | One primary endpoint/ subclinical | No specification | Noninferiority | JT | Not specified |
| *83* | *Tengstrand et al. 2007 (102)* | Disease Activity Score (DAS28), Health Assessment Questionnaire (HAQ) score and bone mineral density of the lumbar spine and hip | Prednisone (Steroids) | Rheumatoid arthritis | Symptom control | Side effects | Outpatient | 58 | 1-2 months | Composite/ both | No specification | Combined | Both | Voluntary foundations |
| *84* | *Thielen et al. 2013 (103)* | Molecular relapse rate at 6 months after discontinuation | Imatinib (Immunosuppressants) | Chronic myeloid leukemia | Cure | Proof of effectiveness | Outpatient | 33 | 3-6 months | One primary endpoint/ subclinical | Superiority | Noninferiority | JT | Voluntary foundations |
| *85* | *Tse W. et al. 2008 (104)* | Change in cognitive, behavioral or motor function | Levodopa (other drugs for chronic conditions) | Parkinsonism | Prognostic improvement | Unclear/little/no benefit | Inpatient | 11 | 3-6 months | Multiple/clinical | both | Combined | Both | Voluntary foundations |
| *86* | *Ulfvarson et al. 2003 (105)* | Montgomery-A ˚sberg depression rating scale (MADRS) | Selective serotonin reuptake inhibitors (Antidepressants) | Elderly patients with no history of depression | Symptom control | Unclear/little/no benefit | Inpatient | 70 | 3-6 months | One primary endpoint/clinical | Noninferiority | Noninferiority | JT | Not specified |
| *87* | *Ungvari et al 1999 (106)* | No primary outcome discernible | Trihexyphenidyl (THP) (Antiparkinsonian drugs)) (Antipsychotics) | Schizophrenia | Symptom control | Side effects | Inpatient | 75 | 3-6 months | Missing | No specification | Combined | Missing | Not specified |
| *88* | *Uretsky et al. 1993 (107)* | 1) treadmill time on maximal exercise testing, 2) distance covered during a 6-min walking test, 3) incidence of treatment failure, and 4) time to treatment failure | Digoxin (Cardiac glycosides) | Mild to Moderate Chronic Congestive Heart Failure | Prognostic improvement | Proof of effectiveness | Outpatient | 88 | Not specified | Multiple/clinical | Superiority | Combined | Both | Voluntary foundations |
| *89* | *Van den Ham et al. 2003 (108)* | Change of Body composition and BMD of the lumbar spine and femoral neck | Prednisone (Steroids) | Renal transplantation patients | Symptom control | Side effects | Not specified | 42 | 3-6 months | One primary endpoint/ subclinical | No specification | Superiority | JW | Not specified |
| *90* | *Van Der Leeden et al. 1986 (109)* | Disease activity, the duration of morning stiffness, the Ritchie index, the number of swollen joints, the ESR according to Westergren, the hemoglobin level, and the Waaler- Rose and latex-fixation tests | Gold (other drugs for chronic conditions) | Rheumatoid arthritis | Symptom control | Unclear/little/no benefit | Outpatient | 24 | Not specified | Multiple/clinical | No specification | Superiority | JT | Not specified |
| *91* | *van Herwaarden et al. 2015 (110)* | Difference in cumulative incidence of major flare | Adalimumab, Etanercept (Immunosuppressants) | Rheumatoid arthritis | Symptom control | Proof of effectiveness | Outpatient | 180 | 1-2 months | One primary endpoint/clinical | Noninferiority | Noninferiority | JT | Not specified |
| *92* | *Van Kraaij et al. 2000 (111)* | Postbrandial systolic blood pressure | Furosemide  Antihypertensive agents | Heart failure (HF) patients | Prognostic improvement | No evidence | Not specified | 32 | 9-12 months | One primary endpoint/ subclinical | No specification | Superiority | JW | Governmental institutions |
| *93* | *Van Kraaij et al. 1999 (112)* | Change on postprandial blood pressure homeostasis | Furosemide (Antihypertensive agents) | Heart failure (postprandial hypertension) | Prognostic improvement | Side effects | Outpatient | 20 | Not specified | One primary endpoint/ subclinical | No specification | Noninferiority | JW | Governmental institutions |
| *94* | *Van Kraaija et al. 2003 (113)* | Requirement to restart or augment furosemide therapy during the 3-month follow-up period | Furosemide (Antihypertensives agents) | Heart failure patients | Symptom control | Unclear/little/no benefit | Outpatient | 29 | 3-6 months | Composite/clinical | Superiority | Noninferiority | JT | Not specified |
| *95* | *van Reekum et al. 2002 (114)* | Behavioral impact of the discontinuation of long- term antipsychotic drugs in institutionalized elderly with dementia | Risperidone, Thioridazine, Loxapine, Perphenazine, Olanzapine, Haloperidol, Nozinan (Antipsychotics) | Dementia | Prognostic improvement | Proof of effectiveness | Inpatient | 34 | 9-12 months | One primary endpoint/clinical | Superiority | Noninferiority | JT | Voluntary foundations |
| *96* | *Vibhagool et al. 2003 (115)* | Episodes of relapse of cryptococcal meningitis | Fluconazole (Infection prophylaxis in chronic disease) | HIV-positive patients with a history of acute cryptococcal meningitis | Prophylaxis | Unclear/little/no benefit | Outpatient | 60 | < 1 months | One primary endpoint/clinical | No specification | Noninferiority | JT | Not specified |
| *97* | *Vilien et al. 2004 (116)* | Relapse | Azathioprine (Immunosuppressants) | Crohn’s disease | Prognostic improvement | Proof of effectiveness | Outpatient | 29 | 3-6 months | One primary endpoint/clinical | No specification | Noninferiority | JT | Voluntary foundations |
| *98* | *Walma et al. 1997 (117)* | Successful withdrawal from diuretic therapy | Furosemide, Chlorothalidone, Hydrochloro­ thiazide plus Triamterene, Epitizide plus Triamterene, and Triamterene (Antihypertensive agents) | Heart failure, hypertension, and non­cardiac ankle oedema | Symptom control | Side effects | Outpatient | 202 | ü3-4 years | One primary endpoint/ discontinuation rate | Superiority | Superiority | JW | Not specified |
| *99* | *Wenzl et al. 2015 (118)* | Time to clinical relapse during follow-up (24 mo) | Azathioprine (Immunosuppressants) | Crohn’s disease | Prognostic improvement | Side effects | Outpatient | 52 | 3-6 months | One primary endpoint/clinical | Superiority | Noninferiority | JT | Pharmaceutical industries |
| *100* | *Wistedt 1981 (119)* | Relapse frequency | Fluphenazine decanoate, Flupenthixol decanoate (Antipsychotics) | Schizophrenia | Prognostic improvement | Proof of effectiveness | Outpatient | 41 | 9-12 months | One primary endpoint/clinical | No specification | Noninferiority | JT | Not specified |
| *101* | *Wolde et al. 1996 (120)* | Flare, defined as recurrence of synovitis | Chloroquine, Hydrochloroquine, parenteral gold, d-Penicillamine, Sulfasalazine, Azathioprine, Methotrexate (Immunosuppressants) | Rheumatoid arthritis | Symptom control | Unclear/little/no benefit | Not specified | 285 | 9-12 months | One primary endpoint/clinical | Superiority | Superiority | JT | Not specified |
| *102* | *Wunderink et al. 2007 (121)* | Relapse rates and social and vocational functioning | Antipsychotic agents (Risperidone, Olanzapine, Quetiapine, Clozapine)  (Antipsychotics) | Schizophrenia or related psychotic disorder | Prognostic improvement | Side effects | Not specified | 131 | < 1-2 years | Multiple/both | No specification | Combined | Both | Governmental institutions,  Voluntary foundations |
| *103* | *Zhu et al. 2008 (122)* | No primary outcome discernible | Mycophenolate mofetil, Methylprednisolone (Immunosuppressants) | Cadaveric renal allograft transplant | Prognostic improvement | Side effects | Outpatient | 45 | < 1-2 years | Missing | No specification | Combined | Missing | Not specified |
